# Improved estimation of hydraulic conductivity by combining stochastically simulated hydrofacies with geophysical data

**DOI:** 10.1038/srep22224

**Published:** 2016-03-01

**Authors:** Lin Zhu, Huili Gong, Yun Chen, Xiaojuan Li, Xiang Chang, Yijiao Cui

**Affiliations:** 1 College of Resource Environment and Tourism, Capital Normal University, Beijing Key Laboratory of Resource Environment and Geographic Information System, Beijing, China; 2 CSIRO Land and Water Laboratory, Canberra, Australia; 3 Beijing Institute of Hydrogeology and Engineering Geology, Beijing, China

## Abstract

Hydraulic conductivity is a major parameter affecting the output accuracy of groundwater flow and transport models. The most commonly used semi-empirical formula for estimating conductivity is Kozeny-Carman equation. However, this method alone does not work well with heterogeneous strata. Two important parameters, grain size and porosity, often show spatial variations at different scales. This study proposes a method for estimating conductivity distributions by combining a stochastic hydrofacies model with geophysical methods. The Markov chain model with transition probability matrix was adopted to re-construct structures of hydrofacies for deriving spatial deposit information. The geophysical and hydro-chemical data were used to estimate the porosity distribution through the Archie’s law. Results show that the stochastic simulated hydrofacies model reflects the sedimentary features with an average model accuracy of 78% in comparison with borehole log data in the Chaobai alluvial fan. The estimated conductivity is reasonable and of the same order of magnitude of the outcomes of the pumping tests. The conductivity distribution is consistent with the sedimentary distributions. This study provides more reliable spatial distributions of the hydraulic parameters for further numerical modeling.

The spatial configuration of hydraulic conductivity (K) is required for accurately predicting groundwater flow and contaminant transport in the subsurface environments^1^,^2^,^3^. As a primary hydrogeologic parameter, K is highly heterogeneous in most alluvial aquifers due to the complex depositional and diagenetic processes of a long-term fan evolution^4^,^5^. Some conventional methods for obtaining the K value include laboratory apparatus (constant-head permeameter and falling-head permeameter)^6^,^7^, *in-situ* pumping tests and slug tests^8^,^9^,^10^. These methods are relatively expensive and can only represent the K in a single piezometer, or within the vicinity of boreholes. That is, the estimated K value is integrated over a specific volume of strata. The difficulty in obtaining representative and sufficient K measurements has hindered our understanding of K spatial variability over a large regional scale.

Geophysical methods are quick, non-invasive, and relatively inexpensive alternatives for aiding hydrogeological studies such as collecting aquifer spatial correlation information^11^,^12^,^13^ and estimating hydraulic properties^10^,^14^,^15^,^16^,^17^. The surface electrical resistivity technique is used to detect resistivity differences within the subsurface. This technique is effective for estimating aquifer parameters related to the pore structure and heterogeneity^18^. Many attempts have been made to construct statistical correlations between conductivity and aquifer resistivity^19^,^20^,^21^,^22^,^23^,^24^,^25^. These semi-empirical correlations are applicable for granular aquifer. The Kozeny-Carman method is the most widely accepted derivations of permeability, which is a function of the medium, including grain size and porosity. However, this method does not work well in heterogeneous strata.

The Markov chain model provides a conceptually simple and theoretically powerful stochastic approach for simulating geological structures with different facies when borehole or geophysical data are sparsely distributed^26^,^27^,^28^,^29^. The continuous Markov chain is described by a mathematical transition probability model with a set of exponential functions. Transition probability models have been used by geologists to describe facies architectures^4^,^27^,^30^, solute dispersion^31^,^32^,^33^, and permeability distributions in hierarchical-scale sediments^34^,^35^. The stochastic simulated hydrofacies reflect a spatial hydro-geological sequence. They can be combined with the Kozeny-Carman method to improve the reliability of the K estimation.

The aim of this study is to improve the accuracy of estimating hydraulic conductivity at a large spatial scale. The proposed approach combines stochastic hydrofacies simulated through transition probability matrix with geophysical data to estimate the K values cell by cell in the three dimensional domain. Spatial data, including stratigraphic, vertical electrical sounding, and salinity data were integrated to get two important parameters (porosity and grain size) required in the Kozeny*-*Carman equation. Observation data of the pumping tests were used to validate estimated K values. An alluvial fan is chosen as a case study area to depict the proposed approach.

The Chaobai River alluvial fan, situated in the northern Beijing Plain, is an important groundwater resource supply region to the Beijing Metropolis. The sedimentary sequences in the study area are highly heterogeneous. The aquifer system can be divided into four zones according to the sedimentary features from north to south (Fig. 1): (1) a single sandy-gravel aquifer (green); (2) a region with two or three sandy-gravel aquifers (light green); (3) multi-aquifers consisting of sandy-gravel and sand with a reduced gravel content (light yellow); and (4) fine-sand multi-aquifers zone (yellow)^5^,^36^. Two sample regions (A and B) located in different areas (Fig. 1) were chosen. Region A with an area of 54 km^2^ belongs to the region of two or three sandy-gravel aquifers. Huairou emergency groundwater resource region (hereafter short for EGRR), built in 2003, is located in Region A. There are 21 groups of pumping wells in the EGRR with the designed groundwater withdrawal of 1.2 × 10^8^ m^3^. Region B with an area of 43 km^2^ belongs to the multi-aquifer system with sandy-gravel to sand, where there are many well-fields. Most of the well-fields were built in 1979 with the original groundwater withdrawal of 1.6 × 10^8^ m^3^. Estimation of the spatial hydraulic conductivity is important for optimal and sustainable usage of the groundwater resource in both regions.

## Results

### Stochastically-simulated hydrofacies

The statistical structure properties (facies volume proportion and mean thickness/length) were derived from the transition probability models based on the stratigraphic data (Table 1). The sub-clay and clay volume proportion in Region B is 0.39, which is twice that in Region A. The gravel volume is dominant in Region A with a proportion of 0.53, which is larger than that in Region B (a proportion of 0.38). The proportions of the medium-coarse sand in the two regions are not significantly different, with a value of about 0.05. In Region A, the mean thickness of gravel is 16.1 m, while that of sub-clay and clay is only 8 m. In Region B, the mean thickness of sub-clay and clay is 13.5 m, whereas the mean thicknesses of the fine sand and medium-coarse sand are smaller than those in Region A. The mean thickness of the gravel is almost the same in Region A and Region B.

The three-dimensional hydrofacies were simulated based on their statistical structural properties. Four boreholes were used to validate the accuracy of the stochastically simulated hydrofacies model. The similarity between the monitoring boreholes and the simulated hydrofacies are 78% and 84% in Region A and 74% and 76% in Region B, respectively. The simulated hydrofacies models in both regions reflect the heterogeneous stratigraphy sequence features (Fig. 2). There is an obvious lithological change from Region A to Region B. The gravel deposit represented by dark green color is dominant and the sub-clay and clay deposit marked by dark yellow is discontinuously distributed in Region A. The interconnection of sand and gravel can facilitates hydraulic links between aquifer systems and can provide pathways for most recharge to the aquifers from the rainfall and river infiltration. The volumetric proportion of gravel deposit decreases sharply in Region B. The connection of the gravel deposit becomes worse in this region since there are more continuous sub-clay and clay layers (Fig. 2), which acts as aquitard layers, and decreases the inter-hydraulic-connection in this area. The stochastic simulated hydrofacies model will provide the grain size information in the three-dimensional domain.

### Conductivity estimation

The hydraulic conductivity of the strata is estimated by combining inversion of the spatial grain size and porosity calculated from the vertical sounding data, and the estimated results are generally consistent with the sedimentary distributions in the three dimensional domain (Fig. 2; Fig. 3). The K values calculated from the Dupuit equation^37^ with two pumping test data (No.1 with the location of X: 348529, Y: 5526914, No.2 with the location of X: 347162, Y: 526250) were used for cross validation. The calculated K at two pumping tests locations are 148 and 134 m/d, whereas the estimated K values under the stochastic framework within the same depths were 102 m/d and 105 m/d, respectively. The K values obtained from two methods are at the same magnitude. Note that the K values calculated from the Dupuit equation and pumping tests represent the average (or uniform) K value of several hydrofacies within the influence radius, while those estimated from the stochastic framework represent the heterogeneous distributions of the hydraulic conductivity.

The K value in Region A generally is greater than that in Region B, especially in the layers at depths greater than 100 m, meaning that the fastest fluid transport for a specified hydraulic gradient occurs at the deep area. In this region the maximum K is located at the northern part of the area at depths of 50 m and 100 m (Fig. 4), while at 150 m depth the larger K values are mainly distributed in the eastern part. In Region B, the distribution of K shows that the areas with lower K are more continuous than those in Region A at the depth of 100 m and 150 m. The estimated spatial K values reflect the real three-dimensional hydraulic property of strata, which can improve the accuracy of the groundwater flow and transport model.

## Discussion

The volumetric proportions of the four hydrofacies at different depths of 50 m, 100 m and 150 m are presented in Table 2. The amount of the gravel deposit increases with the increasing depth, while that of fine sand deposit decreases with the depth in Region A. The gravel volumetric proportion increases from 54.6% at 50 m depth to 68.9% at 150 m depth. The sub-clay and clay content shows a peak at 100 m depth with a value of 19.3%, then, falls to 6.6% at 150 m depth. The proportions of sub-clay and clay and medium-coarse sand both increase with depth in Region B. However, the proportion of gravel decreases with depth. The gravel and sub-clays and clay proportions are 78.3% and 7.4% at the depth of 50 m. The proportion of gravel decreases to 14% at the depth of 150 m, while that of sub-clay clay reaches to 55% (Table 2).

In Region A, with an increase in depth, the mean K values generally increase. Most values are within the range of 50 m/d to 250 m/d, accounting for more than 40% of the total cell number (Table 3). The cell percentage of K values ranging from 50 m/d to 250 m/d at the depth of 100 m is larger than that at the depth of 50 m and 150 m. The amount of the values greater than 500 m/d increases with depth, which reflects the increasing proportion of larger grain-size facies such as gravel. The amount of the values less than 3 m/d decreases with depth, which means the declining proportion of the sub-clay and clay. In Region B the mean K value generally decreases with depth. The mean K values at the depth of 50 m, 100 m and 150 m are 255 m/d, 103 m/d and 99 m/d, respectively. The mean K at 50 m depth in Region B is larger than that in Region A. The main reason may be the difference of lithological proportion. Table 2 shows that the volumetric proportion of gravel at the depth of 50 m is 78% in Region B, which is larger than other layers with different depths in A and B regions. In Region B, the proportions of K higher than 500 m/d decrease with depth. The cells with clay material are dominant in Region B, and the K values are set as a constant low value.

The grain size and porosity are two parameters affecting the estimated hydraulic conductivity (see Eq. 1 in Method section). The original values of the two parameters were varied within a range from −20% to 20%. There are 8 runs for each parameter. Each run represents a value change at a 5% increment. The response of the estimated K shows that the mean K value is positively correlated with grain size and porosity in both A and B regions, and the change of hydraulic conductivity is more sensitive to the change of porosity (Fig. 5). The change of porosity has a significant effect on K value in Region B than that in Region A. However, the change of grain size has the same effect on K value estimation in both regions due to the same grain size setting. For example, the average grain sizes of fine sand and medium-coarse sand are set as 0.2 mm and 0.5 mm in both regions.

We assumed that mean lengths in strike and dip directions are as a ratio of those in vertical direction during the process of estimating the spatial K values. In the future, we will collect multiple sources data reflecting the distribution of the stratigraphic feature to determine the statistic properties in the horizontal direction.

## Methods

Two important components are integrated for estimating the spatial K distributions. One is to determine the grain size from stochastically heterogeneous hydrofacies using the Markov chain method in the three-dimensional domain. The other is to obtain the spatial porosity distributions using Archie’s law on basis of the geophysical data. Borehole stratigraphic data are used for constructing three dimensional hydrofacies. The input data for determining the porosity includes geophysical data and hydro-chemical data. The spatial K values of saturated layers were estimated through the Kozney-Carman equation cell by cell with the determined grain size and porosity under the ArcGIS platform. The constant-rate pumping tests were used to cross-validate the accuracy of estimated K values.

### Data sets

**Figure 1:**
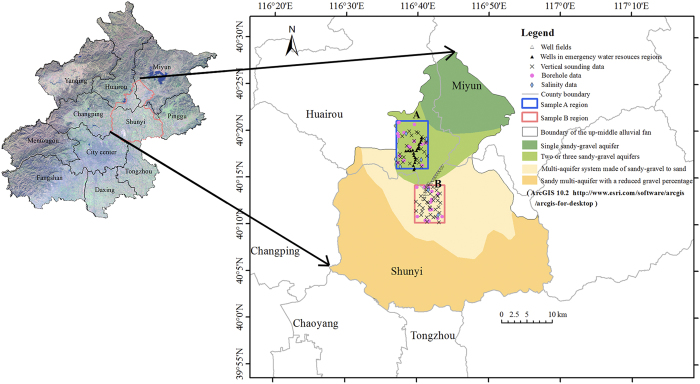
Location of the study area, aquifer lithology and the distribution of the borehole data, the sounding data and salinity data. (Spatial thematic layers including study area boundary, county boundary, field data, aquifer type and Landsat image were overlapped using ArcGIS 10.2 platform. http://www.esri.com/software/arcgis/arcgis-for-desktop).

**Figure 2:**
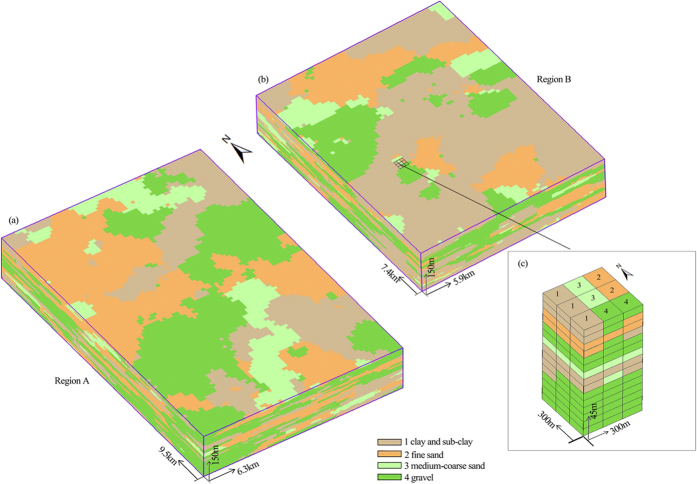
Distribution of the simulated hydrofacies models in Region A (Fig. 2a) and Region B (Fig. 2b) and the detail information of the partial strata in Region B with 45 m depth (Fig. 2c). Figure 2a–c are exaggerated by 10 times in vertical direction.

**Figure 3:**
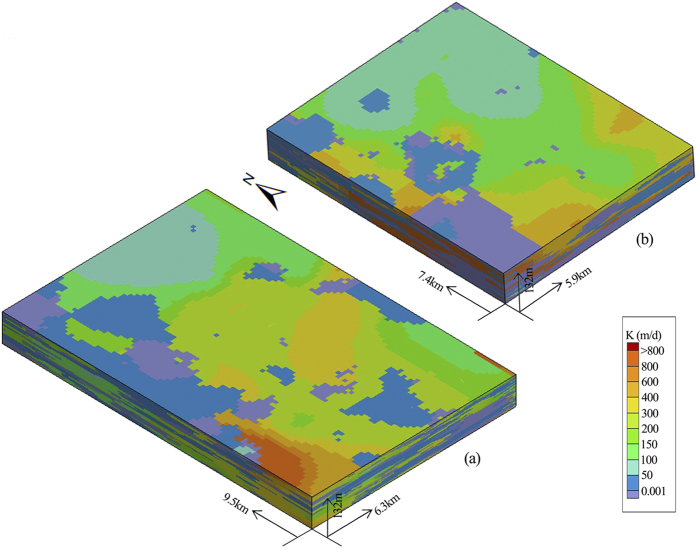
Configuration of K at the strata with depth from 18 m to 150 m in Region A and Region B.

**Figure 4:**
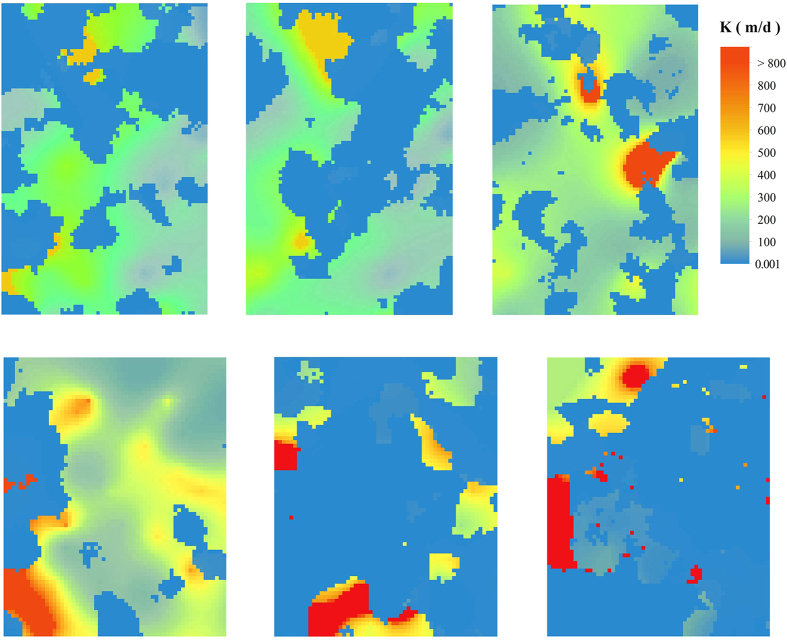
Distribution of K in Region A and Region B. a, b and c are K at the depth of 50 m, 100 m and 150 m in Region A. d, e and f are K at the depth of 50 m, 100 m and 150 m in Region B.

**Figure 5:**
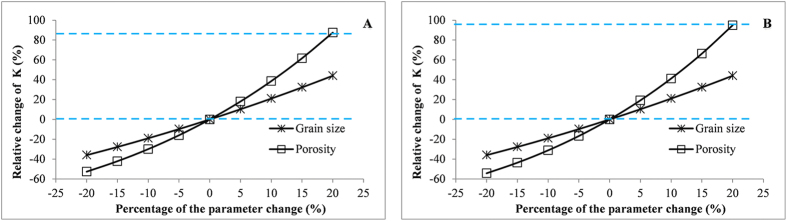
Mean hydraulic conductivity on different grain size and porosity in Region A and Region B.

There are four important data sets for estimating the K values: Stratigraphic, vertical electrical sounding, salinity, and pumping test data. The stratigraphic data are from borehole core descriptions. There are 46 boreholes in Region A and 20 in Region B (Fig. 1). Four hydrofacies (sub-clay and clay, fine sand, medium-coarse sand, and gravel) were classified based on the interpretations of the cores and textural descriptions of the hydrofacies in the boreholes.

Vertical electrical sounding (VES) data were obtained using a Schulumberger array, yielding the apparent electrical resistivity of subsurface aquifer systems. The VES tests were carried out with the semi-distance between current electrodes (AB/2) up to 225 m and the potential electrode (MN) varying from 1 to 30 m. There are 25 VES detecting positions in Region A and 33 positions in Region B (Fig. 1), generally with 12 measurements corresponding to different depth at each detecting position. There are 300 detection points in Region A and 396 points in Region B. The real resistivity of the saturated water-bearing material was needed when applying Archie’s law to calculate the porosity. The VES data were inverted using Occam’s inversion^38^, which is an approach to resolve the regularization of nonlinear inversion problems. This inversion method can fit observed data to a prescribed tolerance. The resulting model has a low root mean square relative error of 2%. The average real resistivity in Region A is larger than that in Region B, and the mean value generally decreases with increasing depth in both regions. The mean resistivity of the strata at the depth of 50 m is about 180 Ω m in Region A and 142 Ω m in Region B, while the mean value at the depth of 150 m decreases to 145 Ω m in Region A and 71 Ω m in Region B.

Seven salinity measurements were obtained throughout the area (Fig. 1). The average salinity concentrations were 596 mg/l and 631 mg/l in regions A and B, respectively. The constant rate pumping tests was used to cross-evaluate the accuracy of estimated K values. The radius of the pumping well is 0.18 m and the total thicknesses of the cumulative pumping layers are 50 m and 53 m according to the detail well description.

### Determination of grain size

The grain size was determined from the stochastic transition probability model. It is a statistical tool for computing probability 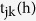 from facies *j* at location *x* transiting to facies *k* at different lag distances *h*. It incorporates geological and sedimentary structural information, including facies spatial correlations, volume proportions and juxtapositional tendencies, into a spatial continuity model^26^. It improves the implementation of geostatistical simulations, especially when field measurements are not sufficiently abundant to support the computation of the spatial continuity model^39^.

The auto- and cross- transition probabilities between different facies in vertical direction were derived from the stratigraphic data. The borehole data are sparsely distributed in horizontal direction, and therefore cannot provide sufficient information of the facies’ correlation structure. Considering that the geological facies distributions are spatially correlated, the Markov chain characteristics (volume proportion and the juxtapositional tendencies) from a transition probability matrix in the vertical direction were assumed to be true in the horizontal direction. The mean lengths in horizontal direction (strike and dip directions) were assumed as a ratio to those in vertical direction^27^,^28^,^40^,^41^. In this study, the ratio is set to 100 times. The T-PROGS (Transition Probability Geostatistical Software)^42^ was adopted to implement the simulation of hydrofacies. In this study, Region A and Region B were discretized with each cell size of 100 m in horizontal direction, and 3 m in vertical direction. The cell size is the same as that in real resistivity distribution.

The average grain size *d*_*(x,y,z)*_ of each cell was determined according to the stochastic simulated hydrofacies. In this paper, the average grain sizes of fine, medium-coarse grain sand and gravel were set as 0.2 mm, 0.5 mm and 2.5 mm based on borehole data.

### Derivation of porosity

The porosity was estimated by using Archie’s law (Eq. 1), which relates the bulk resistivity of granular medium to the porosity of saturated porous deposits. This widely used Archie’s law is valid for clean sand formations^43^. We assumed there is no clay in three facies (gravel, medium-coarse sand and fine sand).


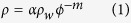


where 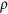 is the bulk resistivity (Ω m), 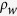 is the fluid resistivity (Ω m), 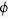 is the medium porosity, 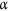
is coefficient associated with the medium intrinsic property and the value range of 0.5≤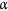 ≤ 2.5, 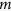 is a cementation factor and has been observed near 1.3 for unconsolidated sands^44^. The coefficients 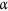 and 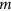 were set as 0.62 and 2.0 in this study. 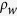 can be calculated through Equation 2^45^,^46^.


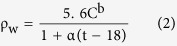


where 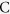 is the concentration of salinity (g/L), t is temperature, b and 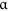 are constant parameters. 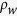 values in Region A and Region B were 9.39 Ω m and 8.89 Ω m with two parameters of b and 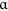 setting as −0.95 and 0.025, respectively. By using the three dimensional real resistivity data, we calculated the porosity of each cell through Equations 1 and 2.

### Estimation of hydraulic conductivity

Once the two important parameters (grain size and porosity) were obtained, the K was estimated from the most commonly used semi-empirical formula, the Kozeny-Carman equation. The Kozeny-Carman equation has taken several forms, including the one below (Eq. 3) after Bear^47^.


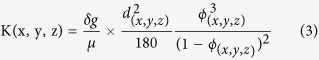


where *d*_*(x,y,z)*_ is the representative grain size diameter (mm) at a spatial point (x,y,z), 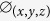 is the porosity, *g* is gravitation acceleration with a value of 9.8 m/s^2^, 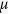 is kinematic viscosity coefficient (kg/m·s) with the value of 0.0014^21^, and 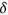 is the density of fluid (kg/m^3^) with the value of 1.0 × 10^3^.

Spatial calculations between the grain sizes and porosities for different layers at different depths were performed by using the spatial function of ArcGIS platform. Considering that the Archie’s law can only be used for clay-free granular sediments, and sub-clay and clay generally has a low K value, this study assumed those cells with the material of sub-clay and clay have a uniform value of 0.001 m/d. The developed new method provide a more reliable spatial distribution of the hydraulic parameters for improved modelling of groundwater flow and transport in the subsurface.

## Additional Information

**How to cite this article**: Zhu, L. *et al.* Improved estimation of hydraulic conductivity by combining stochastically simulated hydrofacies with geophysical data. *Sci. Rep.* **6**, 22224; doi: 10.1038/srep22224 (2016).

## Figures and Tables

**Table 1:** Volumetric proportion and mean length of different hydrofacies.

Lithology type		Sub-clay	Fine sand	Medium-coarse sand	Gravel
Volumetric Proportion	Sample A	0.18	0.24	0.05	0.53
	Sample B	0.39	0.17	0.06	0.38
Lens length in vertical direction (m)	Sample A	8.0	9.0	6.0	16.1
	Sample B	13.5	7.5	5.0	16.0

**Table 2:** Volumetric Proportion of four hydrofacies at different depths.

	Depth (m)	Proportion (%)			
		clay and sub-clay	fine sand	medium-coarse sand	gravel
Sample A	50	4.2	38.3	2.9	54.6
	100	19.3	23.9	1.7	55.1
	150	6.6	21.7	2.7	68.9
Sample B	50	7.4	13.1	1.2	78.3
	100	46.9	27.4	6.9	18.8
	150	55.0	19.2	11.4	14.2

**Table 3:**
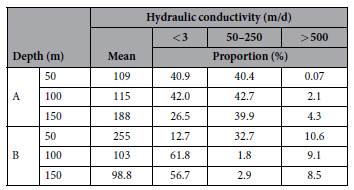
Statistics features of the K values at depths of 50, 100 and 150 m in two sample regions.

^*^The proportion is the ratio of the grid number with specific ranging K values to the total grid number.
